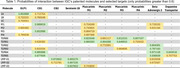# AI in the drug discovery pipeline

**DOI:** 10.1002/alz70861_108629

**Published:** 2025-12-23

**Authors:** Néstor F. González García, Paola Ruiz Puentes, Diego Rodriguez‐Soacha, Daniel Crovo, Ram Mukunda

**Affiliations:** ^1^ IGC Pharma, Bogotá, Bogotá D.C Colombia; ^2^ IGC Pharma, Potomac, MD USA

## Abstract

**Background:**

Scientists spend vast amounts of resources and time to produce safe and effective treatments across a large range of diseases and symptoms. Often, 3 or more years are spent in drug discovery for compound screening and lead optimization. We show an AI model that accelerates the discovery process, thereby allowing researchers to reach clinical phases faster, eliminating years of lab work.

**Methods:**

We used the protein–ligand with adversarial augmentations network (PLA‐Net) to predict target–ligand interactions as a first step in a hybrid in‐silico and in‐vitro pipeline. We extended the target proteins of this model with AD related targets such as dopamine and serotonin receptors and transporters, and muscarinic, oxytocin, cannabinoids, beta‐adrenergic, and GLP1 receptors. We then predicted the interaction of the targets on our patented molecules. The next steps in this process include: testing foundational models of bioactivity and contrastive learning, to identify possible activities of molecules, including some of ours; dynamic molecular docking for some targets of interest in AD; and finally, assays of activity to validate our in‐silico findings on an in‐vitro environment.

**Results:**

We successfully validated the new targets with commercial molecules that have previously known interactions. For example, we tested Semaglutide, which is an effective GLP1 agonist, and the model predicted a 0.9316 probability of the molecule to be an agonist with GLP1. We repeated this process for each target. From there, we tested 4 of our families of drugs (1’s, M’s, TGR’s, and LMP’s) on the trained targets. We identified some families with significant interactions such as the LMP’s with Serotonin‐1B, the TGR’s with beta‐adrenergic‐2, and the 1’s with GLP1. Table 1 has a subset of the results from this first phase.

**Conclusions:**

The results show the promise of an appropriate AI framework to accelerate the discovery of activity between molecules and targets, particularly in receptors with importance to therapeutic treatments for AD. For example, targeting beta‐adrenergic‐2 receptors because of its relation to neurogenesis and synaptic disfunction. Moving to later stages of the proposed pipeline will highlight and validate the potential of our molecules in the field of medicine and AD.